# The Use of the Coenzyme Q_10_ as a Food Supplement in the Management of Fibromyalgia: A Critical Review

**DOI:** 10.3390/antiox11101969

**Published:** 2022-09-30

**Authors:** Luca Campisi, Concettina La Motta

**Affiliations:** Department of Pharmacy, University of Pisa, Via Bonanno 6, 56126 Pisa, Italy

**Keywords:** ubiquinone, ubiquinol, coenzyme Q, CoQ_10_, fibromyalgia, food supplement

## Abstract

The coenzyme Q_10_ is a naturally occurring benzoquinone derivative widely prescribed as a food supplement for different physical conditions and pathologies. This review aims to sum up the key structural and functional characteristics of Q_10_, taking stock of its use in people affected by fibromyalgia. A thorough survey has been conducted, using Pubmed, Scifinder, and ClinicalTrials.gov as the reference research applications and registry database, respectively. Original articles, reviews, and editorials published within the last 15 years, as well as open clinical investigations in the field, if any, were analyzed to point out the lights and shadows of this kind of supplementation as they emerge from the literature.

## 1. Introduction

**Figure 1:**
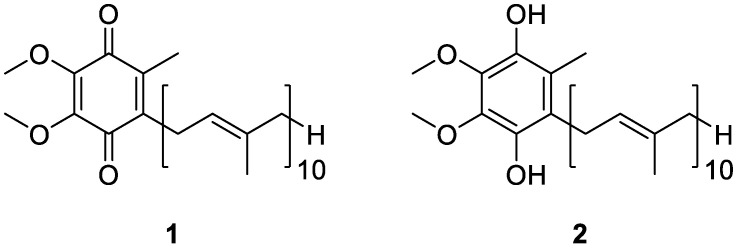
Chemical structures of ubiquinone (**1**) and ubiquinol (**2**).

Coenzyme Q_10_ (CoQ_10_) is a 1,4-benzoquinone derivative that is ubiquitous in nature, existing as a redox pair composed of ubiquinone (**1**, Figure 1) and its reduced form, ubiquinol (**2**, Figure 1). While the former is mainly acknowledged as the key cofactor for mitochondrial enzyme complexes, the latter is claimed for its potent antioxidant properties, and both functions make the compound especially attractive for healthcare providers [1].

CoQ_10_ was first approved in 1976 as a cardiovascular drug for the treatment of heart failure in Japan since it was shown that patients exhibiting heart disease had significantly low levels of CoQ_10_ in both blood and tissue [2]. From then on, and thanks to its favorable combination of functional activity and safety profile, it began to be prescribed for an ever-increasing number of physical conditions and pathologies. Although, it was downgraded to the role of over-the-counter food supplement. Ageing, myopathy, cardiomyopathy, high blood pressure, dyslipidemia, migraine, diabetes, infertility, Friedreich’s ataxia, and neurologic disorders like Parkinson’s and Huntington’s diseases are but a few cases that today prompt practitioners to prescribe CoQ_10_ [3]. To this already extensive list, fibromyalgia has recently been added once it became clear that CoQ_10_ deficiency and mitochondrial dysfunction are both implicated in its pathophysiology [4].

This review aims to sum up the key characteristics of CoQ_10_, taking stock of its use by people affected by fibromyalgia. A thorough survey has been accomplished, using Pubmed, Scifinder, and ClinicalTrials.gov as the reference research applications and registry database, respectively. Original articles, reviews, and editorials published within the last 10 years, as well as open clinical investigations in the field, if any, were analyzed to point out the lights and shadows of this kind of supplementation as they emerge from the literature.

## 2. Fibromyalgia: Key Characteristics and Therapeutic Approaches

Fibromyalgia (FM) is a pathology characterized by chronic and widespread musculoskeletal pain, often associated with asthenia and fatigue and a large set of somatic and neuro-vegetative symptoms. Sleep disorders are also present, characterized by frequent nocturnal awakenings which exacerbate the fatigue condition. In particular, the so-called alpha-delta anomaly is considered to be specific to FM, as once deep sleep is reached, characterized by delta waves at the electroencephalogram, there is a sharp return to superficial sleep, characterized by alpha waves. Cognitive disorders are present in the majority of patients, being related to difficulty in concentration and short-term memory loss [5].

To date, the prevalence of FM is estimated between 5 and 7%, while the incidence level ranges between 7–11 cases per 1000 people per year. It is more frequent in women than in men, with 11 vs. 7 cases per 1000 people, respectively [6,7]. Moreover, it can develop at any age. Actually, a juvenile fibromyalgia syndrome (JFM) is known constituting a complex condition that affects approximately 2–7% of school-aged children showing chronic musculoskeletal and diffuse pain, fatigue, and sleep and mood disturbances [8]. At the same time, FM can also be diagnosed in people at an advanced age. According to some reports, the prevalence of FM reaches a peak between the age of 55–65 years, and the main challenge is a timely and correct diagnosis as patients are frequently identified with rheumatoid arthritis, arthrosis, and rheumatic polymyalgia [9]. Obesity is also commonly associated with FM [10], with a prevalence ranging from 47 to 73% [11]. Stress, depression, anxiety, chronic sleep deprivation, and low physical activity have been linked to an increase in body weight in FM patients. Vincent and co-workers recently assessed the complex relationship between BMI, physical, and psychological factors in these patients but failed to highlight a clear relationship. In fact, they found that with an increase in BMI, it becomes more difficult for the patient to engage in physical activity, leading to a worsening of the symptoms of FM [11].

Currently, the diagnosis of FM is carried out according to the American College of Rheumatology (ACR) 2016 criteria, based on the scores of the widespread pain index (WPI) and the symptom severity scale (SSS) [12]. Mainly based on clinical parameters, these diagnostic criteria frequently overlap with those of other diseases, thus, making FM hard to uncover. In fact, on average, a period of 2.3 years from the first complaint is necessary to get to a definitive diagnosis, excluding those diseases having symptoms, but not causes, common to FM by the evaluation of selected markers.

For some time now, researchers have been calling for specific biomarkers. As thoroughly reviewed by Ablin and co-workers, it should be possible to highlight both a predisposition to FM (from a genetic point of view) and the manifestations of the disease. However, those investigated so far, including serological alterations and instrumental investigations, are for research purposes only [13].

The etiology of FM has not yet been fully understood, and uncertainty still exists concerning the pathophysiological framework. The preeminent hypothesis calls into question a dysregulation in the mechanisms of pain control by the central nervous system (CNS) [14,15,16,17,18,19,20,21,22], while according to other studies, FM would be supported by an inflammation of the small peripheral fibers [23].

Anyhow, FM is clearly characterized by mitochondrial dysfunction [24]. Sprott and co-workers demonstrated a change in the number and size of mitochondria in patients with symptomatic FM [25], and this evidence has been corroborated by additional studies, proving reduced mitochondrial DNA concentrations and CoQ_10_ levels accompanied by the increased expression of pro-inflammatory interleukins IL-1β and IL-18, higher levels of TNF-α [26], and the activation of NLRP3 (NOD-like receptor family, pyrin domain containing 3) and caspase-1. Also, Bullon and co-workers demonstrated that AMPK was not phosphorylated in the fibroblasts of FM patients, and this was in charge of decreased mitochondrial biogenesis, reduced oxygen consumption, decreased antioxidant-enzyme-expression levels, and mitochondrial dysfunction. Moreover, AMPK impairment also results in a marked NLRP3 inflammasome protein activation and a subsequent increase in the serum levels of IL-1β and IL-18 [27,28].

The multifaceted nature of FM requires a multimodal and multidisciplinary approach, mainly aimed at reducing the severity of the symptoms. However, on the basis of the scientific evidence available to date, there are no fully satisfying protocols. Moreover, poor adherence to the therapeutic indications and the presence of comorbidities are two very frequent factors that can modulate the worsening of symptomatology, leading to its chronicity [29,30,31,32].

Over the last decade, the use of personalized dietary regimes has become increasingly important. The supplementation of minerals, such as selenium, zinc, iron, and magnesium, but also vitamins like B9, B12, A, and D, as well as antioxidants, is pursued to complement the nutritional strategy, avoiding nutritional deficits, taking control of oxidative stress, and supporting the immune system [33,34,35,36,37]. Among the plethora of compounds exploited for their antioxidant properties, CoQ_10_ has the preeminent role of supplementing FM patients, as is thoroughly discussed below.

## 3. Coenzyme Q_10_ as a Key Functional Derivative

Coenzyme Q (CoQ) is a widely distributed naturally occurring lipophilic benzoquinone, firstly discovered by Crane and co-workers more than 60 years ago in beef mitochondria [38]. From a chemical point of view, the molecule is characterized by the presence of a quinoid structure, bearing a main side chain comprised of the repetition of several isoprenyl units. As for humans and many mammals, 10 residues are featured in the predominant form of the coenzyme, while other homologous forms are also present. Most of the CoQ_10_ in our body is produced endogenously, but a small amount is also ingested daily through the diet, being mainly present in meat and fish and, in much lower quantities, in some vegetables. Due to its wide prevalence in living organisms, this coenzyme is further referred to as ubiquinone [39].

Since the first pioneering study by Crane and co-workers, the crucial function of CoQ_10_ in the mechanisms of ATP production at the mitochondrial level was deciphered and acknowledged. In particular, the intense research activity carried out by Peter Mitchell, who, thanks to these studies, was awarded the Nobel Prize, clarified CoQ_10_ role as a key cofactor in the oxidative phosphorylation.

**Figure 2:**
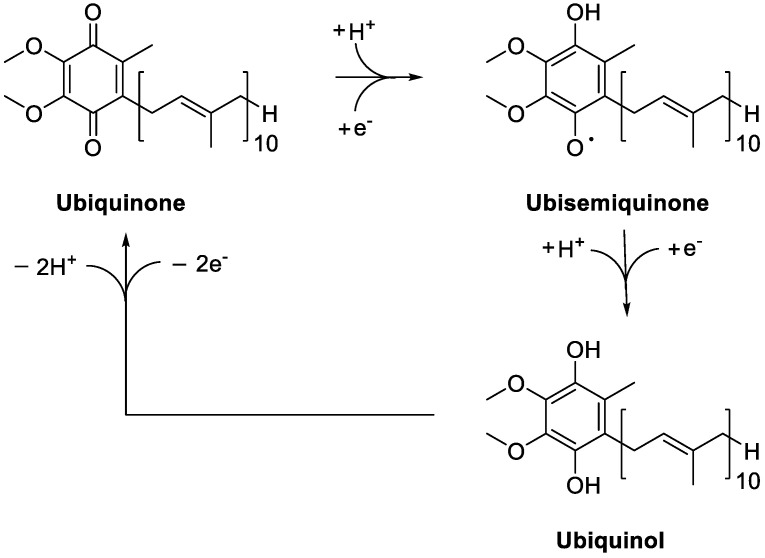
Redox cycling of Coenzime Q_10_.

As thoroughly reviewed by several authors [40,41,42], CoQ_10_ acts as a mobile electron carrier in the electron transfer chain at the inner mitochondrial membrane, but it is also located in blood lipoproteins and cell membranes, where it plays a key antioxidant role once in its reduced form. Indeed, as CoQ_10_H_2_, it is able to quench free radicals and regenerate α-tocopherol and ascorbate [43,44,45]. Several NAD(P)H-dependent reductases ensure the generation of ubiquinol from ubiquinone, possibly via the ubisemiquinone intermediate, depending on the oxidation state of the cofactor (Figure 2). They include NAD(P)H:quinone reductase 1 (NQO1), catalyzing the reduction of the coenzyme to CoQH_2_ through a two-electron reaction, and NADH-cytochrome *b*_5_ reductase and NADPH-cytochrome P450 reductase, which are one-electron reductases [46,47,48,49,50,51,52]. Combined with ubiquinol, these enzymes give rise to the trans-plasma membrane antioxidant system, contributing to the maintenance of the cell antioxidant activities.

In addition to redox activity, more recent data revealed that CoQ_10_ influences the expression of a significant number of genes. Investigated in HeLa cells by Gorelick and co-workers, it was proved to alter 264 sequences, enriching, in particular, lipid-related genes [53]. Using the human intestinal cell line CaCo-2, Groneberg and co-workers demonstrated that CoQ_10_ treatment increases the expression of 694 genes encoding proteins involved in cell signaling, intermediary metabolism, transport, transcription control, disease mutation, phosphorylation, embryonal development, and binding [54,55], thus, confirming the crucial functional role of this compound.

## 4. Sources of Coenzyme Q_10_ and Metabolic Fate

CoQ_10_ is biosynthesized in all tissues and recovered in all membranes. In human tissues, the highest amount of cofactor is found in the heart, liver, kidneys, and muscles, being the districts with either high energy requirements or metabolic activity [56]. At the subcellular level, as demonstrated in rat tissues, CoQ_10_ is mainly localized in Golgi apparatus, lysosomes, and mitochondria [40]. Its amount may vary significantly with respect to the physio-pathological conditions of the individual, gradually reducing as a result of the progression of aging and/or the onset of pathologies [57]. In these cases, supplementation with exogenous CoQ_10_ helps to replenish the poor physiological amount of the coenzyme. The recommended daily intake of CoQ_10_ from exogenous sources ranges from 30–100 mg for healthy people but rises up to 60–1200 mg when it is used as an adjunct supplement in some pathological conditions [58]. In any case, CoQ_10_ is characterized by an estimated no observed adverse effect level (NOAEL) of 1200 mg/kg/day, based on a 52-week chronic toxicity study in rats, and an estimated acceptable daily intake (ADI) of 12 mg/kg/day, ensuring a daily intake without an appreciable health risk throughout life. Furthermore, several studies have shown that, following supplementation, CoQ_10_ does not accumulate in the human body after the cessation of treatment and this does not affect its endogenous synthesis [59,60,61,62].

Two different sources of exogenous CoQ_10_ may be exploited, represented by food matrices and food supplements. While the former is subjected to the variability of the dietetic regimens, the latter assures the constant, regular, and controlled intake of CoQ_10_ over time.

### 4.1. Food Matrices

The distribution of CoQ_10_ among the different food matrices varies significantly, as it is mainly found in meat and fish, while cereals, fruit, and vegetables represent a minority source. The first study to report the CoQ_10_ levels in food matrices was published in Japan in 1986 [63]. As expected, meat and fish turned out to be the richest sources due to their relatively high levels of lipids and mitochondria [64]. However, the coenzyme is not equally distributed among the different tissues of the same animal and varies according to its function (e.g., heart, liver, muscles, etc.) [65,66].

Regarding meat, the highest CoQ_10_ level can be found in reindeer, then beef, pork, and chicken. Despite the differences, meat represents undoubtedly the most important source of CoQ_10_, representing 64% of the total CoQ_10_ intake for Danes [67], 55% for those in Finland [64], and 44% for Japanese people [68]. As for fish, substantial differences in CoQ_10_ content were observed, being the coenzyme notably expressed in horse mackerel and sardine and less represented in eels and salmon, despite their significant fat content. However, the consumption of fish and shellfish is very different around the world, and its impact on the dietary intake of CoQ_10_ ranges from 9% in Northern European countries [64] to 22% in Japan [68]. The concentration of CoQ_10_ in chicken eggs ranges from 1 to 4 mg/kg, while dairy products are very low in CoQ_10_ compared to animal tissues. Regarding products of a plant-based origin, a high CoQ_10_ amount characterizes parsley and spinach. However, the highest CoQ_10_ content may be found in oils. The composition of the oils strictly depends on the plant from which they originate, and the concentration of CoQ_10_ is higher in plants belonging to the Brassicaceae and Fabaceae family [63]. On the contrary, the CoQ_10_ content in rice bran and coconut oil proved to be below the detection limit. Various types of nuts and seeds are also quite rich in CoQ_10_, such as peanuts, sesame seeds, pistachios, walnuts, and hazelnuts, while lower amounts are contained in chestnuts and almonds. In most cereals, the CoQ_10_ content is negligible, with CoQ_9_ being the dominant isoform (4–23 mg/kg) [68]. CoQ_10_ was detected in Japanese millet and buckwheat, while its content in barley and oats was not detected. Soybeans are relatively high in CoQ_10_, but much less CoQ_10_ can be found in their processed versions, such as tofu, soy milk, and yogurt. Most fruits and berries represent a very poor source of CoQ_10_, except avocado, where the CoQ_10_ content, 9.5 mg/kg, is probably linked to its high lipid content. Black currant is another exception, with a CoQ_10_ level of 3.4 mg/kg.

A relationship has been highlighted between the technological processing of food, its fat content, and CoQ_10_ concentration [69]. In this regard, less processed products have higher quantities of CoQ_10_. Actually, the destruction of CoQ_10_ was observed during the boiling process, while a reduction of 14–32% occurred during the frying process. As for fat content, foods with a higher quantity of lipids generally have higher quantities of CoQ_10_. Indeed, fresh whole milk contains 1.2 mg/kg of CoQ_10_, while the amount of CoQ_10_ in UHT milk (having a reduced fat content) is more than halved (0.5 mg/kg) [70]. Similarly, levels of CoQ_10_ are reduced in fermented products such as yogurt, sour milk, and kefir, containing about two-thirds of the CoQ_10_ detectable in milk with the same fat content, while the content is even lower in reduced-fat products. Interestingly, a lower CoQ_10_ content (0.3 mg/kg) was found in yogurt derived from goat and sheep milk despite the much higher fat content. Finally, yogurt that is acknowledged as being fat-free contains negligible concentrations of CoQ_10_.

### 4.2. Food Supplements

Over time, several formulations of CoQ_10_ have been developed for the oral supplementation of the coenzyme. They show different degrees of bioavailability of the target molecule, depending on the used ingredients and the way they are assembled. Among those investigated, the nano-dimensioned forms proved to be the better choice for clinical practice as they allow a significant improvement in the solubilization and bioavailability of CoQ_10_ when compared to their non-nano-dimensioned counterparts [71]. Representative examples are summarized in Table 1 and commented on further in the details below.

Swarnakar and co-workers exploited the polylactic-co-glycolic acid (PLGA) polymer to obtain CoQ_10_-loaded nanoparticles. Investigated in vivo for their pharmacokinetic profile, the loaded nanoparticles showed a sustained CoQ_10_ plasma level for at least 72 h after a single oral administration to Sprague Dawley rats. Significantly, when compared to a non-nanoparticulate formulation, the CoQ_10_-loaded nanoparticles gave an almost four-fold improvement in the oral bioavailability of the coenzyme. To rationalize the observed results, the authors called into question an electrostatic interaction between the positively charged CoQ_10_ nanoparticles and the negatively charged sialic acid of mucin (of the gastrointestinal tract), facilitating the subsequent internalization of the particles through endocytosis [72]. Additional particles, endowed with a high CoQ_10_ encapsulating efficiency, were also obtained by developing nanoscale liposomes. Different mixtures of cholesterol, soy-derived phosphatidylcholine, and alpha-tocopherol were used to achieve the sphere-shaped vesicles, all proving to be effective in stably trapping the target coenzyme [73,74,75]. Zhou and co-workers developed lecithin nanocapsules, obtained by overlaying nanolayers of polymer and enclosing the CoQ_10_. Compared to other formulations, nanocapsules have the added value of protecting the compound while allowing its controlled release from the matrix. Moreover, the authors found that, in the form of nanocapsules, CoQ_10_ has greater bioavailability than common tablets. Actually, once administered orally in mice, there was an increase of 176.6% in the bioavailability of the coenzyme after 24 h [76].

Further attempts to ameliorate the bioavailability of CoQ_10_ by improving its solubility and dissolution rate have been pursued by obtaining solid dispersion [77]. Bhandari and co-workers exploited poloxamer-188 (P188) as the vector, and the resulting binary P188- CoQ_10_ dispersion allowed for an increase in both solubility and in vitro dissolution of CoQ_10_ in water [78]. Also, the solid dispersion obtained by Nepal and co-workers by using poloxamer-407 combined with the hydrophilic fumed silica Aerosil^®^ 200 allowed for an increase in both the solubility and stability of CoQ_10_ [79]. Terao and co-workers complexed CoQ_10_ with the cyclic oligosaccharide γ-cyclodextrin. Administered to healthy adult volunteers under fasting conditions as a single dose containing 30 mg CoQ_10_, the γ-cyclodextrin complex allowed for a significant increase in the plasma levels of CoQ_10_ when compared to the same amount of coenzyme administered as a mixture with microcrystalline cellulose [80].

**Table 1:** Summary of studies investigating CoQ_10_ encapsulation into different delivery systems.

Delivery System	Emulsifier and Additives	Study Findings	Reference
Nanoparticle	Polylactic-co-glycolitic acid	Administered as a single oral dose to Sprague Dawley rats, nanoparticles allowed for a sustained CoQ_10_ plasma level for at least 72 h. An almost four-fold improvement in oral bioavailability of CoQ_10_ was obtained, if compared to non-nanoparticulate formulation.	Swarnakar et al. [72]
Liposome	Cholesterol Soya bean phosphatidylcholine	The effects of temperature, pressure, and components on CoQ_10_ loading in liposomes were investigated and optimized. The optimized conditions allowed for a 82.28% entrapment efficiency of CoQ_10_.	Xia et al. [73]
Liposome	Cholesterol Phosphatidylcholine	The Rapid Expansion of Supercritical Solutions (RESS) technique was used to obtain highly dispersed, nanoscaled CoQ_10_ liposomes. Their CoQ_10_ entrapment efficiency was up to 90%.	Xu et al. [74]
Liposome	Cholesterol Egg phosphatidylcholine 1,2-Distearoyl-sn-glycero-3-phosphoethanolamine-N-[methoxy(polyethyleneglycol)-2000]	Administered by intracoronary infusion to rabbits, with an acute experimental myocardial infarction, they proved to effectively protect the myocardium from ischemia/reperfusion damage by diminishing the size of the irreversibly damaged zone.	Verma et al. [75]
Nanocapsule	Lecithin Caprylic/capric triglycerides Glycerol	Nanocapsules were prepared by high-pressure homogenization. Administered orally in mice, they increased the bioavailability by 176.6% for the coenzyme after 24 h.	Zhou et al. [76]
Solid Dispersion	Poloxamer-188 Water	A simple, rapid, cost effective, uncomplicated, and potentially scalable method was developed, to obtain a binary solid dispersion. It exhibited a remarkably improved aqueous solubility and dissolution of CoQ_10_.	Bhandari et al. [78]
Solid Dispersion	Poloxamer 407 Aerosil^®^ 200	The solid dispersion made from CoQ_10_, poloxamer 407, and Aerosil^®^ 200, used in the weight ratio of 1:5:6, allowed for an increase in both the solubility and dissolution of the coenzyme.	Nepal et al. [79]
Capsule	γ-Cyclodextrin	Administered to healthy adult volunteers as a single dose, capsules containing CoQ_10_ complexed with γ-cyclodextrin allowed for a significant increase in the absorption and bioavailability of the coenzyme.	Terao et al. [80]
Nanoemulsion	Rice bran oil Octenyl succinic anhydride modified starch	A stable nanoemulsion was obtained, showing a CoQ_10_ encapsulation efficiency higher than 98%.	Cheuk et al. [81]
Nanoemulsion	d-α-Tocopheryl polyethylene glycol 1000 succinate Lecithin	Two different nanoemulsions were obtained, using either the water-soluble alpha-tocopheryl polyethylene glycol 1000 succinate (CoQ_10_-NE-TPGS) or lecithin (CoQ_10_-NE-LC) as the key lipid solubilizers. After intravenous administration to Sprague Dawley rats, CoQ_10_-NE-TPGS delivered CoQ_10_ to heart tissue more effectively than CoQ_10_-NE-LC.	Zhou et al. [82]

A number of different nanoemulsions were also developed, all demonstrating an improvement in CoQ_10_ bioavailability. In particular, Cheuk and coworkers dissolved CoQ_10_ in rice bran oil and incorporated the resulting solution into an aqueous octenyl succinic anhydride-modified starch. The high-pressure homogenization of the resulting mixture allowed the workers to obtain a stable nanoemulsion, showing a CoQ_10_ encapsulation efficiency of higher than 98% [81]. Zhou and co-workers used either the water-soluble alpha-tocopheryl polyethylene glycol 1000 succinate (TPGS) or lecithin (LC) as the key lipid solubilizer. Once administered intravenously in male Sprague Dawley rats, they both allowed for the rapid and wide distribution of CoQ_10_ into the tissues. However, the TPGS-based nanoemulsion turned out to be more effective in increasing CoQ_10_ bioavailability when compared to its LC-based counterpart. Significantly, it also guaranteed a more marked CoQ_10_ distribution to the heart tissue. Actually, five minutes after the intravenous administration of the nanoemulsion, the amount of the coenzyme from the TPGS-based system was 2.8-fold higher than that obtained with the LC-based prototype. Moreover, the observed difference in the concentrations remained unchanged over time, although gradually decreasing [82].

When using CoQ_10_ supplementation, the plasma levels of the coenzyme should be monitored to ensure the efficacy of the treatment, especially considering both the different bioavailability between the commercial formulations and the inter-individual variability [83].

CoQ_10_ is generally quantified by liquid chromatography (HPLC) after its extraction from the plasma. The reduced form, CoQ_10_H_2_, is rapidly oxidized to CoQ_10_ by oxygen. Therefore, its determination in plasma samples is rather difficult. The percentage of CoQ_10_H_2_ and CoQ_10_ in plasma varies within the wide 51–96% range [84,85,86,87], and it is assumed that this discrepancy is due to the redox conversion between the two forms before analytical determination. To keep the endogenous concentration of ubiquinol stable, a meticulous blood sample preparation process must be employed, involving the rapid freezing of the plasma at −80 °C. Accordingly, it is not practical to set up the CoQ_10_H_2_ measurement as a routine test. The concentration of CoQ_10_ in plasma is strongly correlated to the circulating lipids and it has been shown that, when measuring the concentration of CoQ_10_, the lipids must, in turn, be considered and the ratio between CoQ_10_ and total or LDL cholesterol calculated.

### 4.3. Metabolic Fate

As a lipophilic substance, CoQ_10_ shares the same fate as lipids. Specifically, the mechanism of CoQ_10_ absorption appears to be similar to that of vitamin E and is enhanced in the presence of a fatty meal. The digestion process facilitates the release of CoQ_10_ from food matrices, while it has no impact on the coenzyme from the different forms of the supplements. After being absorbed in the small intestine through mixed micelles, made up of pancreatic secretions and bile, CoQ_10_ is incorporated into chylomicrons and brought along the lymphatics to the liver for a subsequent redistribution via the bloodstream, mainly within VLDL, LDL, and HDL [88]. Studies in rat models demonstrate that CoQ_10_ is reduced to ubiquinol during or after absorption in the small intestine, and approximately 95% of the circulating CoQ_10_ in humans is represented by the reduced form [89]. Overall, the pharmacokinetic profile of the coenzyme shows a T_max_ ranging from 5.80 to 8.10 h, depending on the characteristics of the used formulation. The observed values testify to slow absorption, typical of hydrophobic and high molecular weight compounds such as CoQ_10_. A second plasma peak may be also observed due to the enterohepatic recycling and redistribution from the liver to the circulation [90]. The distribution of CoQ_10_ in the human body mainly affects tissues with high energy expenditure or metabolic activity, such as the myocardium, kidneys, liver, and muscles, also characterized by a high lipid content. About 40–50% of CoQ_10_ is present in the mitochondrial inner membrane, and small amounts are found in other organelles and also in the cytoplasm. CoQ_10_ is metabolized in all tissues, and the resulting metabolites are phosphorylated in the cells, transported through the plasma, and then excreted by the urine. However, the urinary metabolites of CoQ_10_ represent only a small fraction of CoQ_10_, as the main route of elimination is biliary and fecal.

## 5. Coenzyme Q_10_ as Food Supplement in Fibromyalgia Patients

Besides its antioxidant efficacy, the rationale for the use of CoQ_10_ as a supplement for FM patients resides in the evidence of its reduced levels in these subjects. A growing body of evidence is demonstrating a causal link between CoQ_10_ supplementation and the relief of feeling fatigued in these people [91]. The main outcomes of the studies completed to date are summarized in Table 2.

In details, investigations carried out by Cordero and co-workers proved that the supplementation of 300 mg/day of CoQ_10_ for 40 days and up to 3 months in different groups of women affected by FM showed some clinical improvements. In fact, as assessed by both the fibromyalgia impact questionnaire (FIQ) and the visual analogical scale of pain (VAS), pain, fatigue, morning tiredness, and tender points were significantly reduced (*p* < 0.01). Moreover, CoQ_10_ supplementation significantly ameliorated the headache impact test (HIT-6). At the same time, recovery of inflammation, antioxidant enzymes, mitochondrial biogenesis, and AMPK gene expression levels, associated with phosphorylation of the AMPK activity, were also observed [92,93,94,95,96].

**Table 2:** Summary of studies investigating the effects of CoQ_10_ supplementation on FM patients.

Year of Study	Number of FM Subjects	Study Findings	Reference
2012	20	Decreased levels of CoQ_10_ in SCs and BMCs were observed in FM patients when compared to healthy subjects. 10 patients were supplemented with 300 mg/day CoQ_10_ for 3 months. An enhancement in CoQ_10_ levels in both SCs and BMCs was observed in the supplemented patients, accompanied by a concomitant improvement of symptoms.	Cordero et al. [92]
2012	20	Decreased levels of CoQ_10_, catalase, and ATP, and increased levels of LPO in BMCs were observed in FM patients when compared to healthy subjects. 10 patients were supplemented with 300 mg/day CoQ_10_ for 3 months. Oral supplementation restored biochemical parameters and induced a significant improvement in clinical and headache symptoms	Cordero et al. [93]
2012	1	Decreased expression levels of mitochondrial chain proteins (complex I, 39-kDa subunit; complex III, core 1 subunit; and complex IV, cyclooxygenase-2) and decreased levels of CoQ_10_ were observed in the BMCs of the patient. Instead, LPO and expression levels of OGG-1 turned out to be increased in BMCs. Supplementation of 300 mg/day CoQ_10_ for 3 months restored the mitochondrial proteins expression levels and CoQ10 levels, decreasing also LPO and OGG-1 expression levels in BMCs. A significant improvement of clinical symptoms was also observed.	Cordero et al. [94]
2013	20	40 days of CoQ_10_ supplementation, 300 mg/day, allowed for a reduction in pain, fatigue, and morning tiredness in FM patients. Furthermore, a recovery of inflammation, antioxidant enzymes, mitochondrial biogenesis, and AMPK gene expression levels, associated with phosphorylation of the AMPK activity, were also observed.	Cordero et al. [95]
2013	4	9 months of CoQ_10_ supplementation, 300 mg/day, allowed for an improvement in clinical symptoms, including pain, fatigue, sleep, and tender points. Somatization and anxiety were also significantly reduced.	Alcocer-Gómez et al. [96]
2017	22	DDM Chinone^®^, containing 200 mg/dose of CoQ_10_ enriched with vitamins E, B2, B6, B12 and folic acid, was administered twice a day for three months. Supplementation exerted beneficial effects in counteracting pain, fatigue, sleep disturbance, and mental difficulties in the treated patients.	Di Pierro et al. [97]
2022	23	Supplementation with CoQ_10_, tryptophan, and magnesium for 3 months proved to be well tolerated by the administered patients. Moreover, it improved physical symptoms such as fatigue, sleep quality and functional capacity, as well as the global well-being.	Gómez-Centeno et al. [98]
2013	10	Patients with juvenile FM displayed significantly increased levels of free cholesterol, cholesterol esters and free fatty acids, accompanied by CoQ_10_ deficiency and increased oxidative stress. Supplementation of 100 mg/day of ubiquinol-10 for 12 weeks increased CoQ_10_ levels and decreased plasma levels of free cholesterol and cholesterol esters. Moreover, chronic fatigue was also improved.	Miyamae et al. [99]
2019	11	Administration of CoQ_10_ to pregabalin-treated FM patients reduced pain, anxiety, brain activity, mitochondrial oxidative stress, and inflammation. Moreover, it also increased the levels of reduced glutathione and superoxide dismutase.	Sawaddiruk et al. [100]

Note. SCs: salivary cells; BMCs: blood mononuclear cells; LPO, lipid peroxidation; OGG-1: oxoguanyne glycosilase 1; FIQ, Fibromyalgia Impact Questionnaire.

Di Pierro and co-workers proved the efficacy of oral CoQ_10_ supplementation, enriched with B2, B6, B9, B12, and E (DDM Quinone^®^) vitamins in 22 female patients (53 ± 9.1 years old) affected by FM who received 400 mg/day of CoQ_10_ for 3 months. The randomized, open-label, cross-over study demonstrated that this supplementation improved most pain-related outcomes by 24–37%, including fatigue (by ~22%) and sleep disturbance (by ~33%) [97].

Gómez-Centeno and co-workers combined CoQ_10_ with magnesium and tryptophan and investigated the effects of this kind of supplementation in a sample of female FM patients aged 18–90 years within the pilot prospective study FATMIA. After 3 months of treatment, a certain improvement in fatigue, sleep quality, and functional capacity was achieved, although the observed differences were not statistically significant. Surprisingly, emotional factors, like depression and anxiety, were not affected, despite the concomitant use of coping strategies and the demonstrated efficacy on the mood of the components of the supplement [98].

Miyamae and co-workers evaluated the efficacy of ubiquinol-10 supplementation in young patients affected by juvenile FM. In the study, 75 patients (13.1 ± 2.45 years old) were randomly assigned to either the intervention group, administered with 100 mg/day of supplement or the control group, administered with a placebo. After 3 months of treatment, the Chalder fatigue scale was used to assess the fatigue status of the participants, who showed a significant improvement (*p* < 0.05) in the fatigue scores [99].

A benefit in CoQ_10_ supplementation was also observed in pregabalin-treated FM patients. In fact, Sawaddiruk and co-workers carried out a double-blind, randomized, placebo-controlled trial, administering 300 mg/die CoQ_10_ for 40 days to a small number of patients in therapy with 150 mg/die pregabalin. The outcomes observed were compared to those resulting from the control group, receiving pregabalin and placebo. Supplementation with CoQ_10_ was proven to reduce pain, anxiety, and brain activity. Moreover, it also decreased mitochondrial oxidative stress and inflammation [100].

Regardless of the FM conditions, the effects of CoQ_10_ supplementation in relieving fatigue were also investigated in healthy subjects.

In 2008, Mizuno and co-workers carried out a randomized study to evaluate the effects of CoQ_10_ administration during physical fatigue. In particular, 17 healthy volunteers (37.5 ± 9.9 years) randomly received 100 or 300 mg/day of CoQ_10_ or a placebo during 8 days of physical activity resulting in the induction of fatigue. Subjective fatigue, measured by the visual analog scale (VAS), turned out to be significantly reduced (*p* < 0.01) in the group treated with 300 mg CoQ_10_ when compared to the placebo group [101].

On the contrary, other investigations failed to demonstrate any efficacy against the same endpoint. Actually, Lee and co-workers carried out a 3-month study on 51 obese subjects with a body mass index (BMI) of ≥25 kg/m, who were randomly assigned to receive either 200 mg/day of CoQ_10_ or a placebo. When compared to the placebo group, the intervention group did not show any significant reduction in fatigue (*p* = 0.28), as assessed through the questionnaire on the severity of fatigue scale (FSS). Besides, the authors did not find any evidence that CoQ_10_ supplementation ameliorated metabolic parameters or inflammatory markers [102]. Similar outcomes were also obtained with 15 randomized sedentary men who received 100 mg/day of CoQ_10_ or a placebo for 2 months. The corresponding index did not show any beneficial effect (*p* > 0.05) in reducing their fatigue during exercise [103].

Therefore, on the basis of the experimental evidence acquired so far, a causal link between CoQ_10_ supplementation and the relief of feeling fatigued can be seen only in FM conditions, offering the prospect of using this compound for the management of people affected by this pathology.

## 6. Conclusions

FM is a complex syndrome for which the pathophysiological framework is still being defined. Over the last decade, the nutritional status of people affected by FM has been the object of thorough research, putting into evidence the key nutritional deficiencies concerning particular minerals, including Se, Zn, and Fe, but also vitamins and compounds endowed with antioxidant properties, like CoQ_10_. As for the latter, low levels of the coenzyme have generally emerged as a common feature in FM patients, thus, opening the way to its reintegration via the use of suitable supplements. The number of investigations aiming to demonstrate the effectiveness of this prescription has increased over time, outlining a positive causal relationship between the amount of CoQ_10_ supplementation and the relief of FM symptoms, the first and foremost being the feeling of fatigue.

According to the outcomes obtained so far, CoQ_10_ might be reasonably regarded as the gold standard supplement for people affected by FM. However, a closer look at the available data highlights the lack of a sound and clear scientific basis for justifying this trumpeted claim. Actually, most, but not all, of the authors highlighted the reduced levels of CoQ_10_ in FM patients; therefore, the consensus of the scientific community on this condition is not unanimous. In particular, Thorsteinsdóttir and co-workers observed no significant differences in the coenzyme content in both the blood and muscle of FM patients when compared to healthy subjects [104]. Although being a single piece of evidence, this still undermines the rationale of CoQ_10_ supplementation. Moreover, scientific investigations that claim the functional efficacy of this supplementation suffer from clear and obvious limits. In particular, besides the biased readings of the experimental outcomes, the number of patients enrolled in the studies is often limited, even consisting of a single patient [94]; this is inadequate evidence that cannot be expanded to the whole FM population. Therefore, prospective and randomized trials, with hundreds of patients per study group, are now required to corroborate the efficacy of CoQ_10_ in the management of FM observed in pilot studies.
